# Supplementary material for: Structural and Dynamic-Based Characterization of the Recognition Patterns of E7 and TRP-2 Epitopes by MHC Class I Receptors through Computational Approaches
Source: Int J Mol Sci. 2024 Jan 23;25(3):1384. doi: 10.3390/ijms25031384 (PMC10855917; doi:10.3390/ijms25031384)
Supplement: Supplementary file 1 [file ijms-25-01384-s001.zip › ijms-2814148-supplementary.pdf]

SUPPLEMENTARY MATERIAL

Structural and dynamic-based characterization of the recognition patterns of E7 and TRP-2 epitopes by MHC class I receptors through computational approaches.

Nicole Balasco[1], Maria Tagliamonte[2], Luigi Buonaguro[2], Luigi Vitagliano[3] and Antonella Paladino[3*]

1 Institute of Molecular Biology and Pathology IBPM-CNR c/o Dep. Chemistry, Sapienza University of Rome, Piazzale Aldo Moro 5, Rome 00185, Italy; nicole.balasco@cnr.it

2 Immunological Models Lab, Istituto Nazionale Tumori - Istituto di Ricovero e Cura a Carattere Scientifico (IRCCS) - "Fond. G. Pascale", via Mariano Semmola 53, 80131 Napoli, Italy; m.tagliamonte@istitutotumori.na.it; l.buonaguro@istitutotumori.na.it;

3 Institute of Biostructures and Bioimaging IBB-CNR, via Pietro Castellino 111, 80131 Napoli, Italy; luigi.vitagliano@cnr.it; antonella.paladino@cnr.it

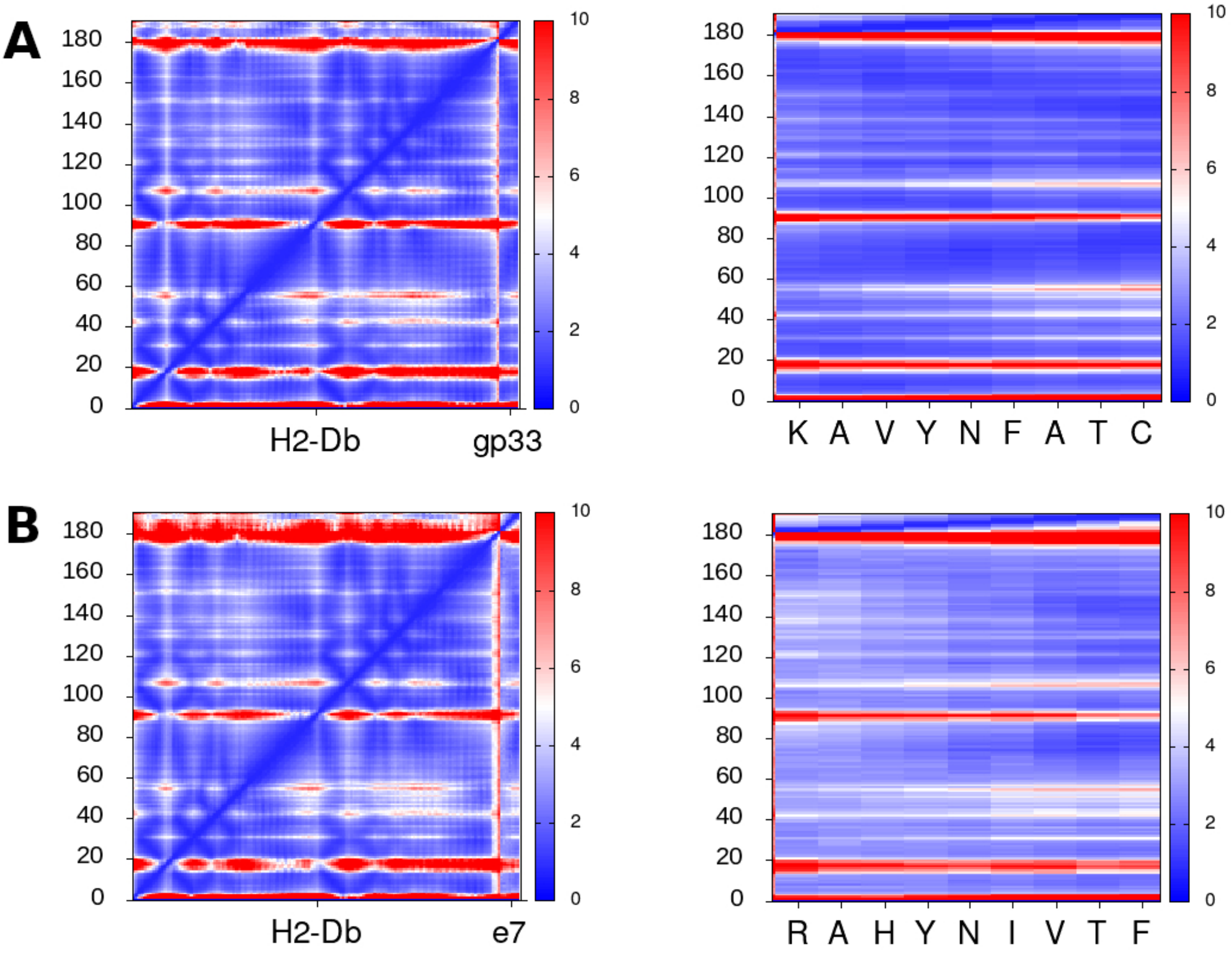

Figure S1. PAE matrices of the AlphaFold first best ranked structural complexes, considered in the present study. Full-length complex is reported and labeled in the left panel whereas a zoom-in of the GP33 (A) and E7 (B) sequences is plotted in the right panel.

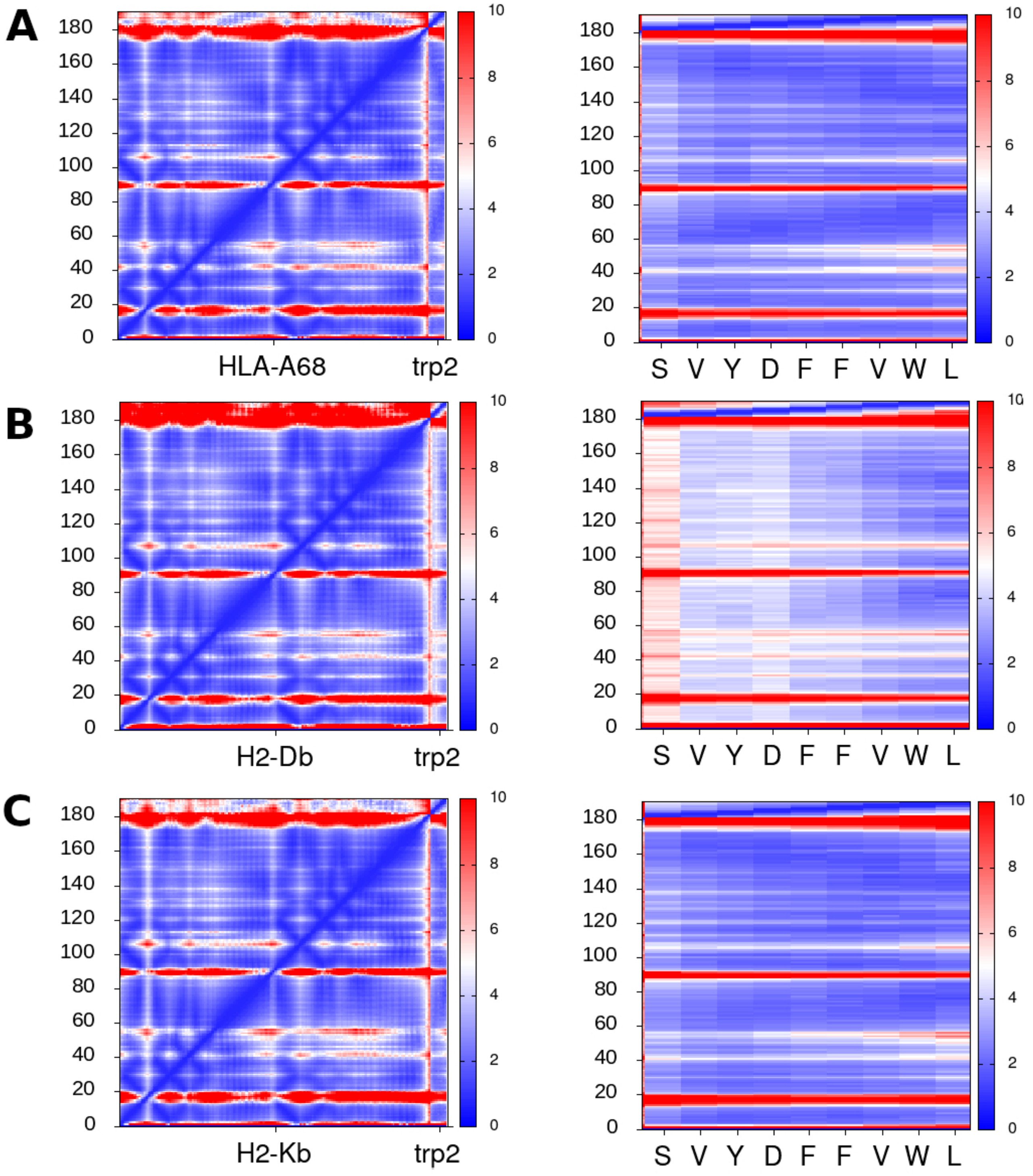

Figure S2. PAE matrices of the AlphaFold first best ranked structural complexes, considered in the present study. Full-length complexes are reported and labeled in the left panel whereas a zoom-in of the TRP2 sequence is plotted in the right panel.

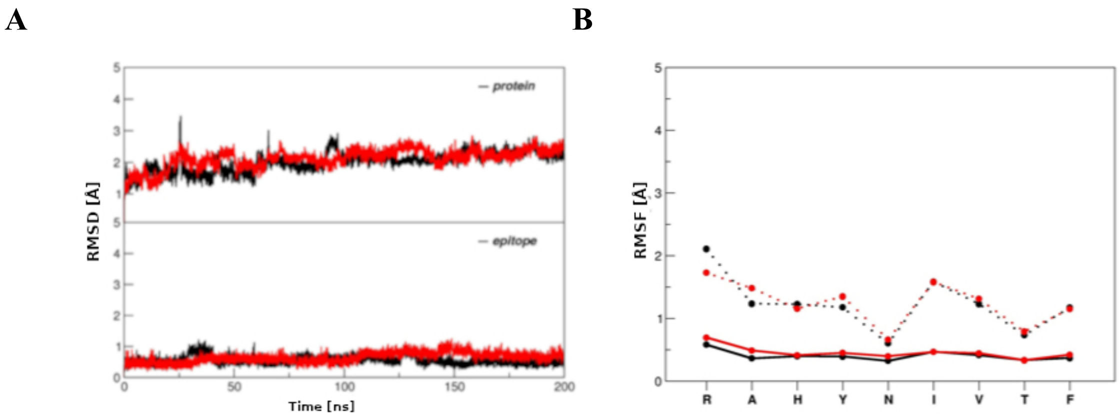

Figure S3. Time evolution of E7^{Db} RMSD values computed against the starting structure in the simulations (run1 in black, run2 in red) (A) and RMSF values of the E7^{Db} epitope amino acid residues of (B). Values have been calculated on the Cα (continuous line) and the side chain (dotted line) atoms.

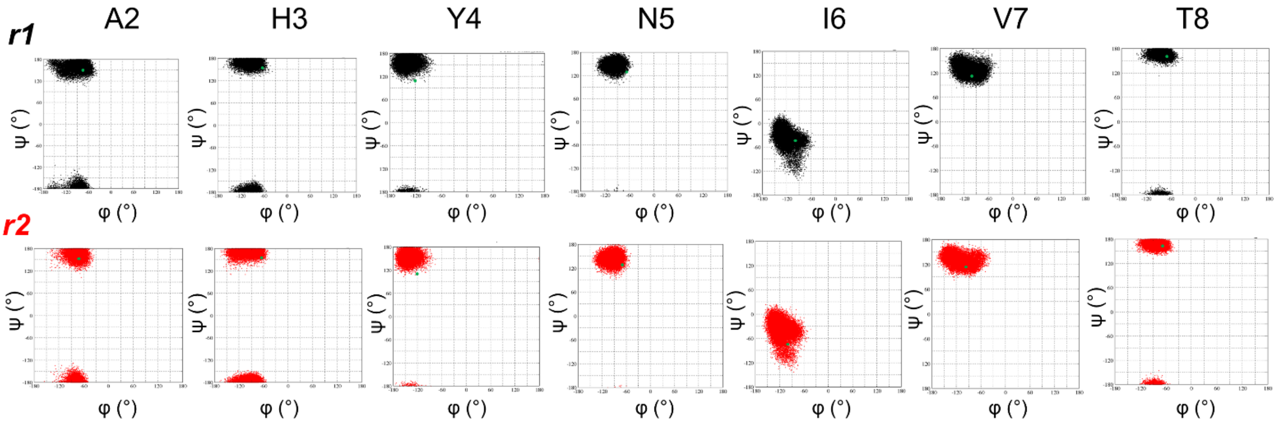

Figure S4. Ramachandran plots of the E7 amino acid residues in the MD simulations (run1 in black, run2 in red). The (φ, ψ) values of the starting structure are indicated by a green dot. Notice that no dihedral distributions can be reported for terminal R1 and F9 amino acids.

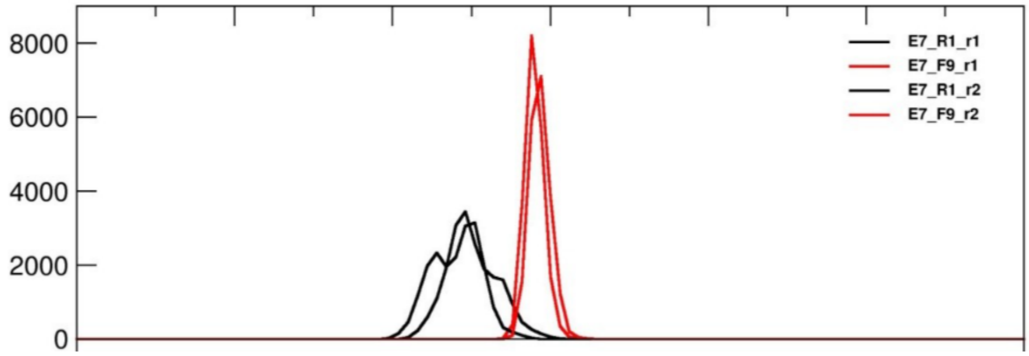

Figure S5. Distance between the center of mass of the terminal E7^{Db} epitope residues R1 and F9 (all atoms) and MHC protein in the simulations.

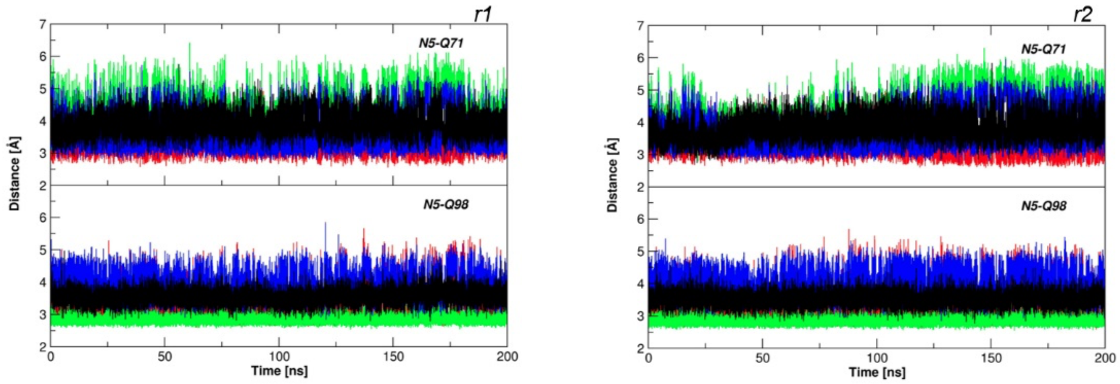

Figure S6. N5 anchoring at MHC cavity. Hydrogen bonds with Q71 (α1) and Q98 (β5). Time-dependent interactions between Oδ1 (black/red) and Nδ2 (green/blue) of N5 from the epitope and Oε1 and Nδ2 of Q71/Q98 from MHC are reported for the two runs.

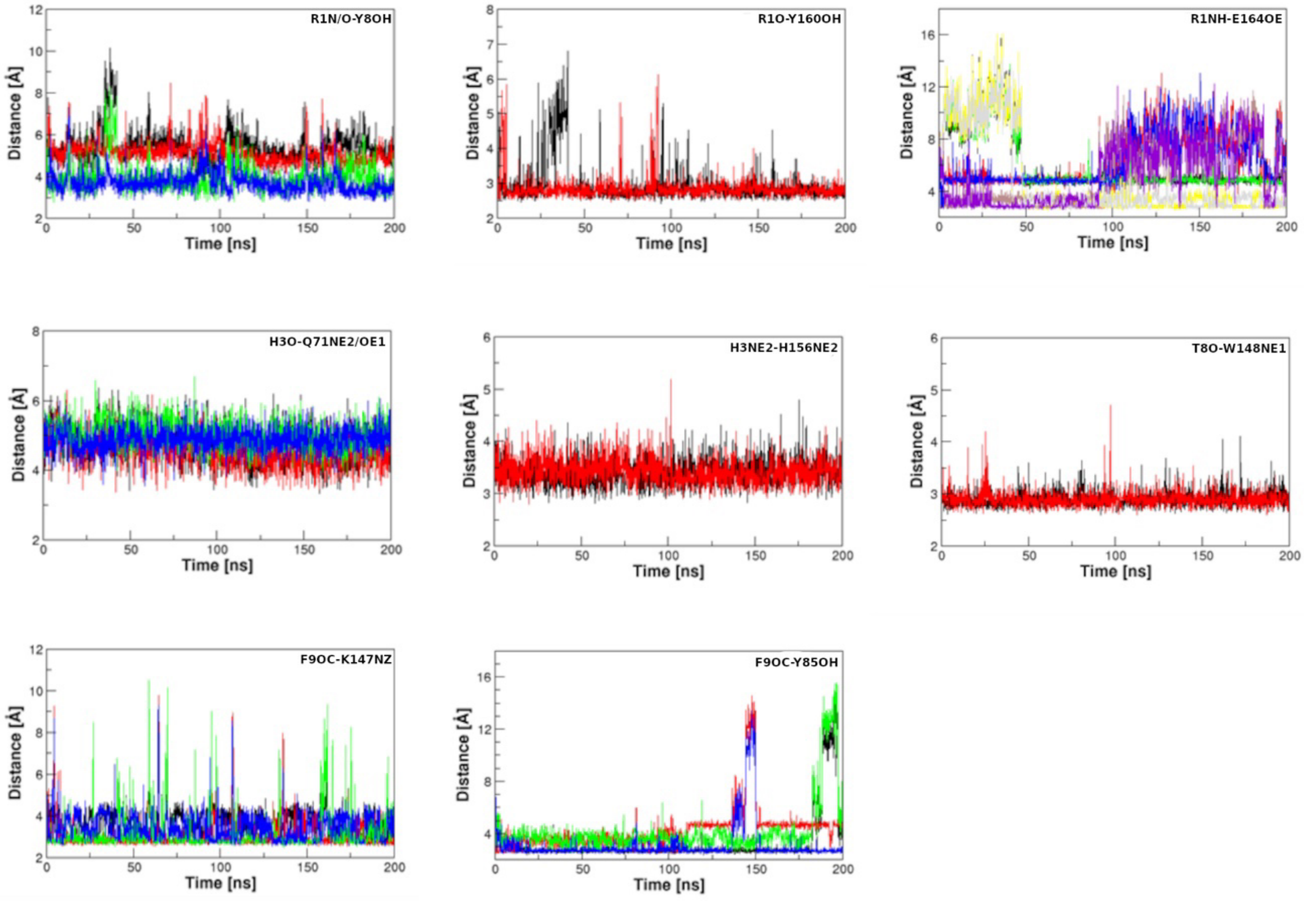

Figure S7: Time-evolution of stabilizing H-bond interactions in $E7^{Db}$ along the simulation time.

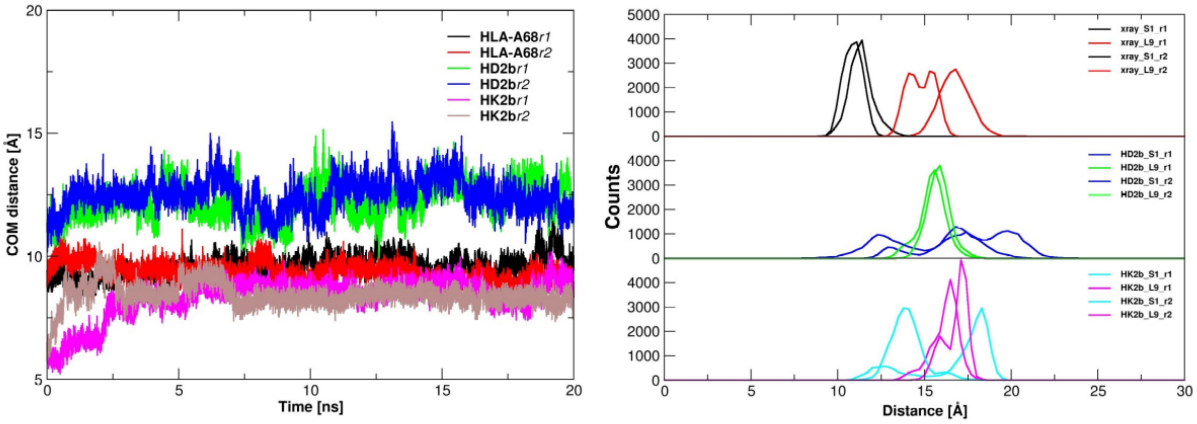

Figure S8. Distance between the center of mass of the terminal epitope residues and MHC protein in the simulations of TRP-2A68, TRP-2Db and TRP-2Kb.

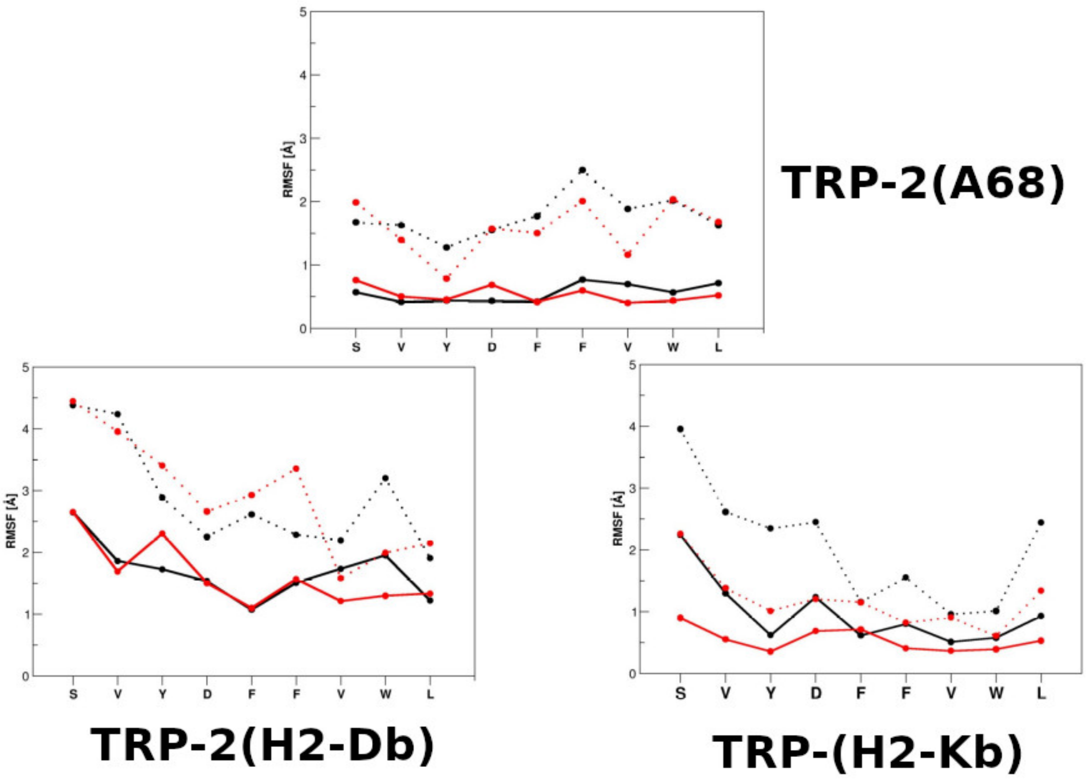

Figure S9. RMSF values of the epitope amino acid residues of TRP-2. Values have been calculated on the Cα (continuous line) and the side chain (dotted line) atoms.

A

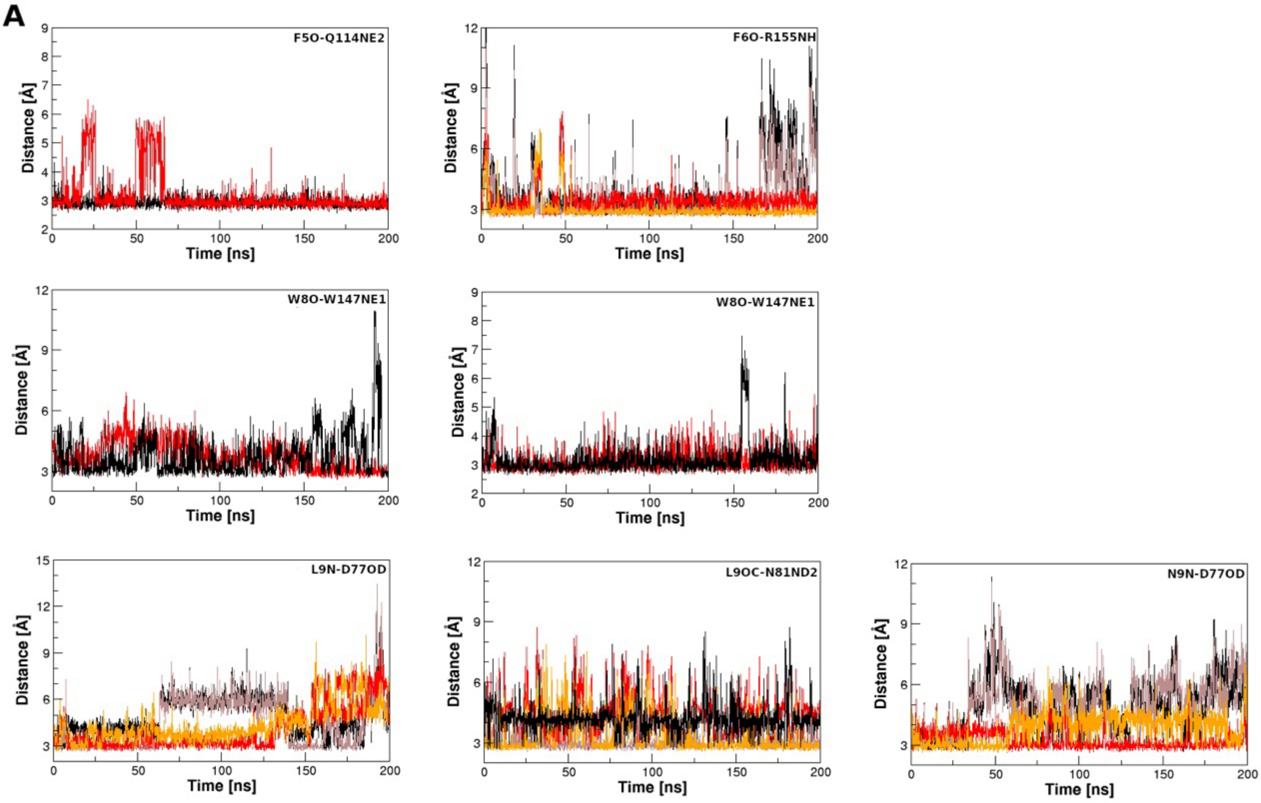

B

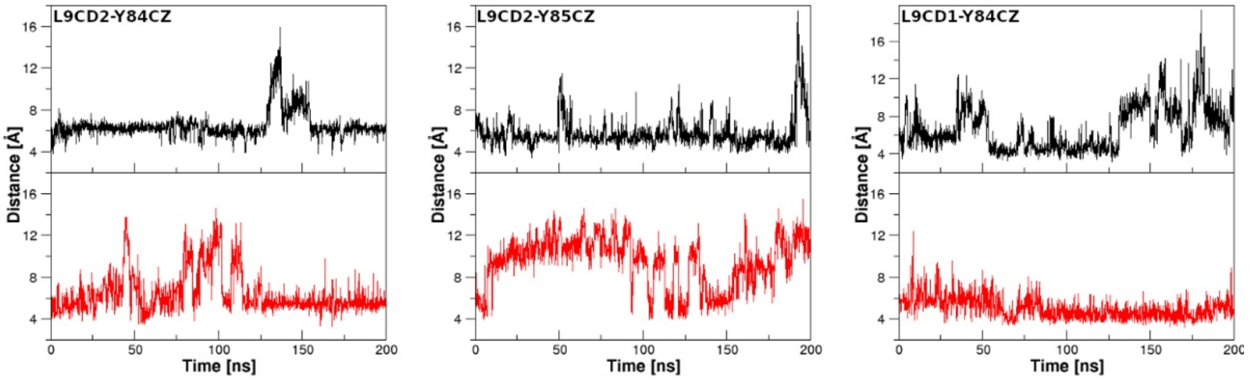

Figure S10. TRP2^{Db} stabilizing interactions. (A) Time-evolution of representative H-bonds in TRP2-MHC as reported in Table 3 of the main text. From top left: bonds mediated by F5 and F6 in H2-K^b; W8 in HLA-A68 and H2-K^b, L9 in HLA-A68, H2-D^b and H2-K^b. (B) Time-evolution of nonpolar interactions mediated by the L9 residue at the C-terminus in H2-D^b (left), H2-K^b (middle) and HLA-A68 (right).

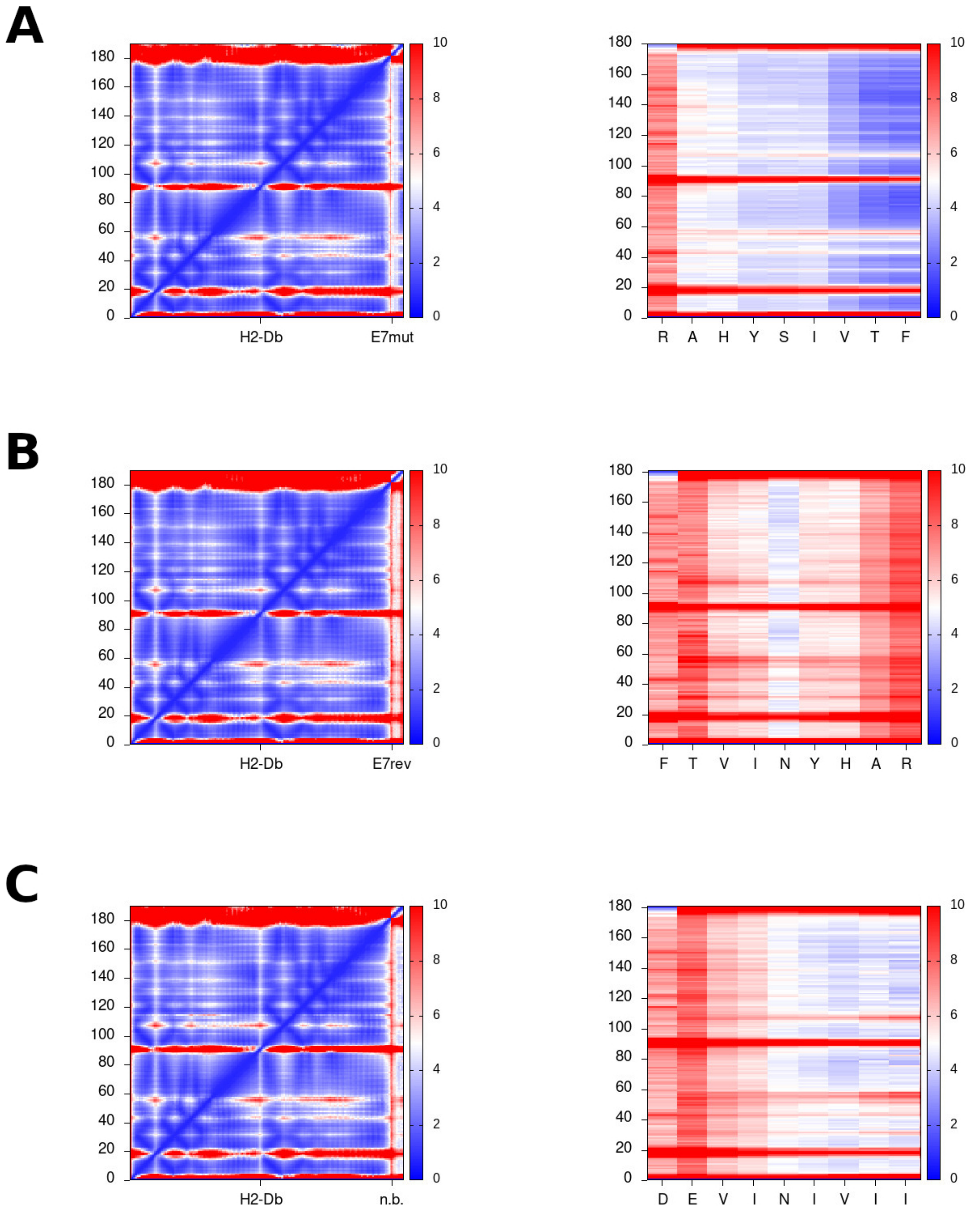

Figure S11. PAE matrices of the best ranked AF models for poor H2-D^b binders. (A) mutant complex E7$_{mut}$-H2D^b (N53S); (B) E7$_{rev}$-H2D^b, with of the E7 epitope sequence in the reverse order; (C) example of non-binder (n.b.) H2D^b epitope from literature data [1] .

Table S1. Structural epitope-H2-D^b complexes retrieved from Protein Data Bank. The table reports all structures of MHC sharing a sequence identity with at least 85% with the H2Db.

PDB ID	Peptide sequence	length	coverage (%)	UniProtKB ID	Sequence Identity	Resolution (Å)
2zok	ABA-SLWNGPHL	9	75	P01899	100	2.10
7jwi	ASNENMETM	9	100	P01899	100	3.02
1hoc	ASNENMETM	9	75	P01899	100	2.40
1ce6	FAPGNYPAL	9	75	P01899	100	2.90
2ve6	FAPGNY-PRQ-AL	9	76	P01899	100	2.65
1bz9	FAPGVFPYM	9	75	P01899	100	2.80
1fg2	KAVYNFATC	9	77	P01899	100	2.75
1s7u	KAVYNFATM	9	93	P01899	100	2.20
1n5a	KAVYNFATM	9	76	P01899	100	2.85
1ffn	KAVYNFATM	9	75	P01899	100	2.70
3buy	LSLRNPILV	9	75	P01899	100	2.60
1n3n	SALQNAASIA	10	77	P01899	100	3.00
6wzy	SGPDNGAVAV	10	76	P01899	100	1.50
5wlg	SQLLNAKYL	9	76	P01899	100	2.10
7n5c	SSLCNFRAYV	10	76	P01899	100	1.87
1juf	SSVIGVWYL	9	75	P01899	100	2.00
1inq	SSVVGVWYL	9	75	P01899	100	2.20
4hv8	ASNENIETM	9	77	P01899	99.64	2.00
4hux	ASNENMETM	9	77	P01899	99.64	2.20
4iho	EGPRNQDWL	9	76	P01899	99.64	2.80
7n5q	SSLCNFRAYV	10	77	P01899	99.64	1.76
2cii	YPAL	4	75	P01899	99.64	2.55
6h6h	SGPSNTPPEI	10	93	Q31167	99.11	2.40
6l9m	SPSYVYHQF	9	76	P01897	93.88	2.60
1ldp	APAAAAAAM/QLSPFP	9	75	P01897	93.75	3.10
1ld9	YPNVNIHNF	9	74	P01897	93.66	2.40
8d5n	HPGSVNEFDFGCGGS	15	75	P01897	93.09	1.80
1rjy	SSIEFARL	8	77	P01901	85.71	1.90
3e6f	IGPGRAFYA	9	75	P01900	85.66	2.41
5kd7	IGPGRAFYV	9	75	P01900	85.66	2.35
5wes	RGPGC	5	75	P01900	85.66	2.71
3dmm	RGPGRAFVTI	10	75	P01900	85.66	2.60
1qo3	RGPGRAFVTI	10	75	P01900	85.66	2.30
2clv	SQYYYNSL	8	77	P01901	85.66	1.90
8d5e	TGAASFDEF	9	75	P01900	85.66	2.46
1t0n	SSIEFARL	8	76	P01901	85.61	1.80
1lek	EQYKFYSV	8	75	P01901	85.4	2.15
1mwa	EQYKFYSV	8	75	P01901	85.4	2.40
1fzj	RGYVYQGL	8	75	P01901	85.4	1.90
1fzm	RGYVYQGL	8	75	P01901	85.4	1.80
3e6h	IGPGRAFYTI	10	75	P01900	85.29	2.10
4hkj	SIINFEKL	8	77	P01901	85	3.00
7ji2	SIIQFEHL	8	77	P01901	85	1.95
6npr	RGPGRAFVTI	10	75	P01900	84.93	2.37
4pg9	FAPGNYPAL	9	76	P01901	84.89	2.40
1nan	INFDFNTI	8	76	P01901	84.89	2.30
1kj3	KVITFIDL	8	76	P01901	84.89	2.30
1kj2	KVITFIDL	8	76	P01901	84.84	2.71
3p4m	YTVKYPNL	8	76	P01901	84.84	2.50
1n59	AVYNFATM	8	76	P01901	84.78	2.95
1fo0	INFDFNTI	8	75	P01901	84.73	2.50
1nam	RGYVYQGL	8	75	P01901	84.73	2.70
1leg	EQYKFYSV	8	75	P01901	84.67	1.75
1bqh	RGYVYQGL	8	75	P01901	84.67	2.80
5j6g	VGITNVDL	8	77	P01898	84.59	3.30
3tid	AVYNFATM	8	76	P01901	84.53	1.65
2mha	RGYVYQGL	8	74	P01901	84.44	2.50
3rgv	WIYVYRPMGCGGS	13	75	P01901	84.36	2.90
1ddh	RGPGRAFVTI	10	75	P01900	84.19	3.10
4hs3	AVYNFATM	8	76	P01901	84.06	2.10
1bii	RGPGRAFVTI	10	100	P01900	83.84	2.40
8d5j	KYLQVASHV	9	75	P01902	83.64	1.95
8d5k	KYRQVASHV	9	75	P01902	83.64	2.07
1k8d	ILMEHIHKL	9	75	P14429	83.58	2.30
1vgk	SYVNTNMGL	9	75	P01902	83.58	2.06
5gr7	YYSIIPHSI	9	75	P01902	83.58	2.40
5j6h	VGITNVDL	8	80	P01898	83.45	2.30
4wdi	LYLVCGERG	9	76	P01902	83.33	2.31
5trz	YQSGLSIVM	9	75	P01902	83.27	2.25
1s7q	AVYNFATM	8	93	P01901	83.19	1.99
2fwo	TYQRTRALV	9	77	P01902	82.98	2.60
3fol	VNDIFERI	8	75	D2YW38	82.48	2.50
1zt1	FEANGNLI	8	76	P04223	81.52	2.50
1zt7	SEFLLEKRI	9	75	P04223	81.45	3.00

Green cells represent structural complexes of H2-D^b receptors (sequence identity > 99% compared to UniProtKB P01899). Dark green is used for the reference structure (GP33-H2-D^b, PDB entry 1fg2).

Table S2. Structural alignment of the peptide-H2D^b X-ray complexes from Table S1.

PDB id	EPITOPE POSITION								
	1	2	3	4	5	6	7	8	9
1fg2	K	A	V	Y	N	F	A	T	C
2zok	Aba	S	L	W	N	G	P	H	L
1hoc	A	S	N	E	N	M	E	T	M
2ve6	K	A	P	G	N	Y	Prq	A	L
1bz9	F	A	P	G	V	F	P	Y	M
1s7u	K	A	V	Y	N	F	A	T	M
3buy	L	S	L	R	N	P	I	L	V
1n3n	S	A	L	Q	N	AA	S	I	A
6wzy	S	G	P	D	N	G	AV	A	V
5wlg	S	Q	L	L	N	A	K	Y	L
1juf	S	S	V	I	G	V	W	Y	L
1inq	S	S	V	V	G	V	W	Y	L
4hv8	A	S	N	E	N	I	E	T	M
4hux	A	S	N	E	N	M	E	T	M
4iho	E	G	P	R	N	Q	D	W	L
7n5q	S	S	L	C	N	F	RA	Y	V
2cii						Y	P	A	L
6h6h	S	G	P	S	N	T	PP	E	I

In green the reference structure (GP33-H2-D^b, PDB entry 1fg2) is indicated. Epitopes sequences used for structural alignment are reported using the IN-OUT scheme shown below where underscored letters indicate amino acids pointing into the H2-D^b binding cavity.

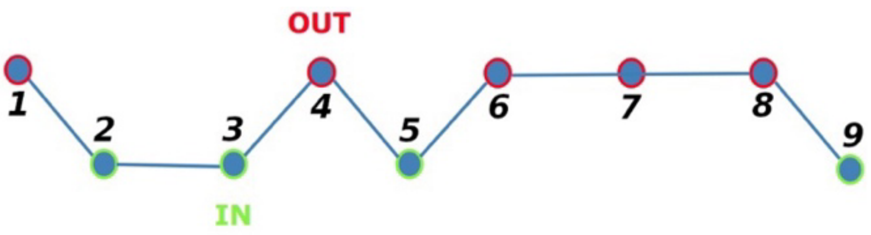

References

1. Hudrisier, D.; Mazarguil, H.; Laval, F.; Oldstone, M.B.A.; Gairin, J.E. Binding of Viral Antigens to Major Histocompatibility Complex Class I H-2Db Molecules Is Controlled by Dominant Negative Elements at Peptide Non-Anchor Residues. *J. Biol. Chem.* **1996**, *271*, 17829–17836, doi:10.1074/jbc.271.30.17829.
